# Crizotinib with or without an EGFR-TKI in treating EGFR-mutant NSCLC patients with acquired MET amplification after failure of EGFR-TKI therapy: a multicenter retrospective study

**DOI:** 10.1186/s12967-019-1803-9

**Published:** 2019-02-21

**Authors:** Wenxian Wang, Hong Wang, Peihua Lu, Zongyang Yu, Chunwei Xu, Wu Zhuang, Zhengbo Song

**Affiliations:** 1 Department of Medical Oncology, Zhejiang Cancer Hospital, Hangzhou, 310022 China; 2 Department of Lung Cancer, The Fifth Medical Center, General of PLA, Beijing, 100071 China; 3 Department of Oncology, Wuxi People Hospital, Wuxi, 214023 China; 4 Department of Oncology, Fuzhou General Hospital of Nanjing Military Command, Fuzhou, 350025 China; 5 Department of Pathology, Fujian Cancer Hospital, Fuzhou, 362200 China; 6 Department of Chemotherapy, Zhejiang Cancer Hospital, East Banshan Road, Hangzhou, 310022 China

**Keywords:** MET amplification, Lung cancer, EGFR, Resistance, Crizotinib, Survival

## Abstract

**Background:**

MET amplification is associated with acquired resistance to first-generation epidermal growth factor receptor (EGFR)-tyrosine kinase inhibitors (TKIs) in treating non-small-cell lung cancer (NSCLC); however, the therapeutic strategy in these patients is undefined. Herein we report the clinical outcomes of patients with c-MET amplification resistance to EGFR-TKIs treated with crizotinib.

**Methods:**

We retrospectively analyzed advanced NSCLC patients from five sites who were diagnosed with EGFR-mutant NSCLC and received EGFR-TKI treatment. After disease progression, these patients were confirmed to have a MET-to-centromere ratio (MET:CEN) ≥ 1.8 based on fluorescence in situ hybridization (FISH) examination and without a T790M mutation. We assessed the efficacy and safety of crizotinib to overcome EGFR-TKI resistance in EGFR-activating mutations NSCLC with acquired MET amplification.

**Results:**

Amplification of the acquired MET gene was identified in 18 patients with EGFR-mutant NSCLC. Fourteen patients received crizotinib treatment after acquired resistance to EGFR-TKIs. Among the 14 patients, 6 (42.9%) received crizotinib plus EGFR-TKI and 8 (57.1%) received crizotinib monotherapy. The overall objective response rate (ORR) and disease control rate (DCR) were 50.0% (7/14) and 85.7% (12/14), respectively. The median PFS (mPFS) of patients receiving crizotinib monotherapy and crizotinib plus EGFR-TKI was 6.0 and 12.6 months, respectively (P = 0.315). Notably, treatment efficacy was more pronounced in patients with crizotinib than patients with chemotherapy (24.0 months vs. 12.0 months, P = 0.046). The mOS for 8 of 14 patients receiving crizotinib monotherapy and 6 of 14 patients receiving crizotinib plus EGFR-TKI was 17.2 and 24.0 months, respectively (P = 0.862). Among the 14 patients, 1 who received crizotinib monotherapy (grade 3 nausea) and 2 who received crizotinib plus EGFR-TKI (grade 3 elevated liver aminotransferase levels) received reduced doses of crizotinib (200 mg twice daily) to better tolerate the dose.

**Conclusions:**

We observed the clinical evidence of efficacy generated by combination of crizotinib and previous EGFR-TKIs after the resistance to first-generation EGFR-TKIs. These results might increase evidence of more effective therapeutic strategies for NSCLC treatment. Combination therapy did not increase the frequency of adverse reactions.

## Background

Lung cancer is one of the most common causes of death from cancer worldwide [1]. Over the past decades, advances in molecular analysis and targeted therapies have evolved the treatment and survival rates for patients with lung cancer. Epidermal growth factor receptor (EGFR) is one of the major driving mutations in non-small cell lung cancer (NSCLC) [2].

EGFR tyrosine kinase inhibitors (TKIs) have been used as first-line treatment of advanced NSCLC patients harboring EGFR mutations, which successfully improved the response rate (RR) and prolonged survival compared with chemotherapy [3–7]; however, with first-generation EGFR-TKIs, most patients exhibit disease progression with a median progression-free survival (mPFS) ranging from 9 to 13 months. Various mechanisms for the acquisition of resistance to EGFR-TKIs have been identified, among which approximately 50% of cases involving acquired resistance (AR) are due to a secondary T790M mutation in exon 20 of EGFR gene [8]. Other causes of AR include bypassing signaling activation, phenotype transition, and aberrant downstream signaling pathways [9–13]. One example of bypassing signaling activation is acquired mesenchymal epithelial transition factor receptor (Met) gene amplification [14], causing c-Met amplification to circumvent the inhibited EGFR phosphorylation kinase pathway and activate downstream signal transduction, such as the PI3K/AKT pathway, thus developing resistance to EGFR-TKI and facilitating tumor cell proliferation. It has been reported that 5%–20% patients harbor c-Met amplifications with acquired resistance to EGFR-TKIs [14–16].

As a MET inhibitor, the efficiency of crizotinib in treating NSCLC patients with MET gene mutations has been demonstrated [17, 18]. The efficacy of combining EGFR-TKIs with MET inhibitors, such as crizotinib, capmatinib, INC280, or savolitinib, has been determined [19]; however, few clinical trials with a large sample size have compared the efficacy of crizotinib monotherapy and crizotinib combined with EGFR-TKIs as a therapeutic strategy and prognosis of EGFR-mutated patients with acquired c-Met amplification. In the present multicenter retrospective study, we analyzed the characteristics of EGFR-mutant NSCLC patients with acquired MET amplifications and the outcomes of crizotinib monotherapy and crizotinib plus EGFR-TKIs after failure of previous lines of EGFR-TKI treatment.

## Materials and methods

### Study cohort

We conducted this multicenter, retrospective study involving five hospitals in China between January 2014 and March 2017. Pathological sub-classification was determined according to the 2015 WHO histologic classification scheme. All patients were confirmed to have advanced or recurrent stage IV NSCLC with EGFR mutations according to the TNM classification (version 7) with an Eastern Cooperative Oncology Group performance status (ECOG PS) of 0–2. The patients all received first-generation EGFR-TKI treatment, then acquired resistance to EGFR-TKI (gefitinib, erlotinib, or icotinib) therapy. The clinical criteria for acquired resistance to EGFR TKIs proposed by Jackman et al. [20] were followed to determine the acquired resistance to a previous EGFR-TKI. Tissue samples (drug-resistant specimens) collected from primary tumors or metastatic sites have been collected after development of resistance against EGFR-TKIs. Then, the tumor tissues were subjected to next generation sequencing (NGS) when the patients agreed. We excluded the patients who had acquired EGFR T790M mutations. When the clinician identified an acquired MET amplification without a T790M mutation, an adequate specimen and FISH was performed for confirmation. The protocol has been approved by the Institutional Ethics Committee at each investigation site.

### EGFR mutation analysis

All tumor tissue samples had routine pathological evaluations to confirm the diagnosis of NSCLC. Tumor tissue (drug-resistant specimens) DNA was extracted from formalin-fixed, paraffin-embedded (FFPE) specimens. All tumor samples were routinely assessed by sectioning, hematoxylin–eosin staining, and visualization under a microscope to ensure tumor content by two pathologists. FFPE sections and smear slides were deparaffinized in xylene and rehydrated in descending grades of absolute ethanol. DNA was extracted using an AmoyDx FFPE DNA Kit (Amoy Diagnostics, Xiamen, China) according to the manufacturer’s instructions.

A Human EGFR Gene Mutation (exons 18–21) Fluorescence Polymerase Chain Reaction (PCR) Diagnostic Kit (Amoy Diagnostics), which was based on ARMS technology, was used to analyze the DNA from the tissue samples. An ADx EGFR Mutations Detection Kit (Amoy Diagnostics) has received China Food and Drug Administration (CFDA) approval for clinical usage since 2010. We defined a cut-off of 2% tumor cell content as a sample quality check according to the minimum requirement of ARMS technology (approximately 1% analytical sensitivity). Samples below this threshold were rejected.

### MET FISH assay

Deparaffinized sections (4–6 μm thick) were subjected to dual-color FISH testing using a MET/CEN7q Dual Color FISH Probe (Vysis, Abbott Molecular, Des Plaines, IL, USA). The MET amplification status was classified into the following 4 categories: negative (MET/CEN7 ratio < 1.8); low (≥ 1.8 ≤ 2.2); intermediate (> 2.2 < 5.0); and high level (> 5.0).

### Treatment

All patients received a standard dose of a EGFR-TKI, such as gefitinib (250 mg per day orally), erlotinib (150 mg per day orally), and icotinib (125 mg 3 times per day orally). Patients received oral crizotinib (250 mg twice daily). Safety assessments included physical examinations, documentation of adverse events, and routine laboratory tests, including hematology (hemoglobin concentration, platelet count, and white blood cell count), chemistry (alanine aminotransferase [ALT], aspartate aminotransferase [AST], alkaline phosphatase, and lactate dehydrogenase), coagulation (prothrombin time and activated partial thromboplastin time), and urinalysis. Adverse events were graded according to NCI CTCAE (version 3.0). The dosage of crizotinib was reduced to 200 mg twice daily or stopped based on adverse reactions.

### Response evaluation criteria

Tumor responses were evaluated every 6–8 weeks in accordance with the Response Evaluation Criteria in Solid Tumors guidelines (version 1.1) to monitor objective tumor responses, including complete response (CR), partial response (PR), stable disease (SD), and progressive disease (PD). The disease control rate (DCR) was defined as the sum of the objective response and stabilization rates (CR + PR + SD). Adverse effects were evaluated according to the Common Terminology Criteria for Adverse Events (CTCAE [v. 5.0]).

### Follow-up evaluation

Progression-free survival 1 (PFS1) was defined as the period from the initial date of EGFR-TKI treatment to the date of confirmation of disease progression. PFS2 was defined as the period from the date of initiating crizotinib treatment to the date of disease progression evaluated by RECIST (version 1.1) or death. Overall survival (OS) was measured from the date of the crizotinib treatment or chemotherapy (after acquired resistance to EGFR-TKI) to death or last follow-up visit. The last follow-up was on May 30, 2018.

### Statistical analysis

Kaplan–Meier estimates and the log-rank test were applied to evaluate PFS and OS. The statistical analysis was performed using SPSS (version 19.0; SPSS, Inc., Chicago, IL, USA). Two-sided P values < 0.05 were considered a statistically significant difference.

## Results

### Characteristics

Eighteen NSCLC patients with EGFR mutations were identified to have c-MET amplification who developed acquired resistance to a first-generation EGFR-TKI, including 11 (61.1%) female patients and 7 (38.9%) male patients. The median age was 57 years (range 37–81 years). Histologically, most patients (16/18 [88.9%]) were diagnosed with adenocarcinomas. Twelve patients (66.7%) were non-smokers. All patients had stage IV NSCLC according to the 7th edition of the AJCC Cancer Staging TNM Staging Guide. Two (11.1%) and 16 patients (88.9%) had exon 19 and 21 L858R mutations, respectively. Twelve (66.7%) and 6 patients (33.3%) received EGFR-TKIs as first- and later-line treatment, respectively. Due to acquired resistance to EGFR-TKIs, 14 patients (77.8%) received crizotinib therapy and four patients received chemotherapy. The patient characteristics are summarized in Table 1.

**Table 1:** Clinicopathologic features among 18 patients for acquired MET amplification

Characteristics	Number	Percentage (%)
*Gender*		
Male	7	38.9
Female	11	61.1
*Age (years)*		
Mean	57 years	
< 60	10	55.6
≥ 60	8	44.4
*ECOG PS*		
0–1	18	100
2	0	0
*Histologic type*		
Adenocarcinoma	16	88.9
Other	2	11.1
*Smoking status*		
No	12	66.7
Yes	6	33.3
*Type of EGFR mutation*		
19 deletion	2	11.1
21L858R	16	88.9
*Type of EGFR-TKIs*		
Gefitinib	10	55.6
Elrotinib	3	16.7
Icotinib	5	27.8
*Treatment lines of EGFR-TKI*		
First line	12	66.7
Second line or more	6	33.3
*Met amplification*		
> 5	12	66.7
2.2–5	4	22.2
1.8–2.2	2	11.1
*Receiving crizotinib*		
Yes	14	77.8
No	4	22.2

### Met amplification and T790M mutation

Among the 18 patients, all tested negative for EGFR T790M mutations. Twelve patients had high MET amplification; four patients had intermediate and two patients had low MET amplification.

### Treatment efficacy

The efficiency of the first-generation EGFE-TKIs was evaluated for 18 patients; 14 patients (77.8%) had PR and four patients (22.2%) had SD. The median PFS1 was 10.5 months (95% CI, 8.91–12.09 months).

After developing acquired resistance to EGFR-TKIs and confirming acquired MET amplification, 14 patients were treated with crizotinib and four patients received chemotherapy. Among the 14 patients who were treated with crizotinib, eight (57.1%) received crizotinib monotherapy and six (42.9%) received crizotinib plus EGFT-TKI treatment. Regarding treatment efficacy in these 14 patients, 7 (50.0%) had PR, 5 (35.7%) had SD, and two (14.3%) had PD. The objective response rate (ORR) and disease control rate (DCR) were 50.0% (7/14) and 85.7% (12/14), respectively. The ORRs were 50.0% and 50.0% for patients treated with crizotinib with or without EGFR-TKIs; no significant difference was detected (P = 1.000). Similarly, the DCRs were not significantly different between the patients treated with crizotinib with or without EGFR-TKIs (87.5% vs. 85.7%, respectively; P = 1.000).

**Fig. 1:**
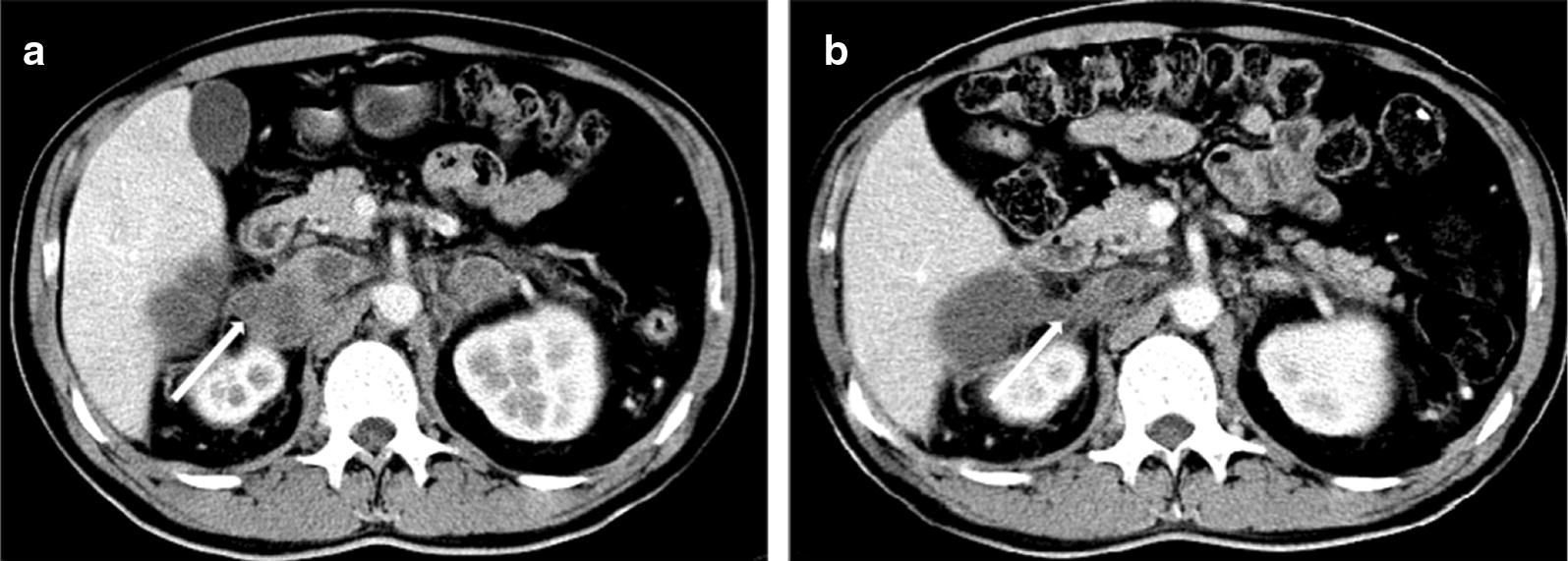
Representative CT scanning result of one patient with acquired MET amplification who responded to crizotinib monotherapy. **a** Before treatment; **b** partial response was detected after 1 month of treatment

**Fig. 2:**
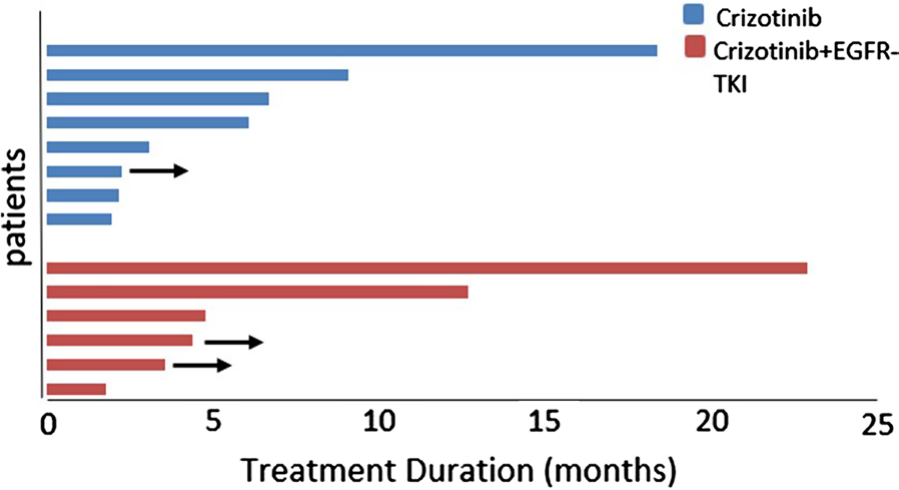
Duration of response with crizotinib treatment in 14 patients. Arrow indicates continuation to crizotinib treatment

**Fig. 3:**
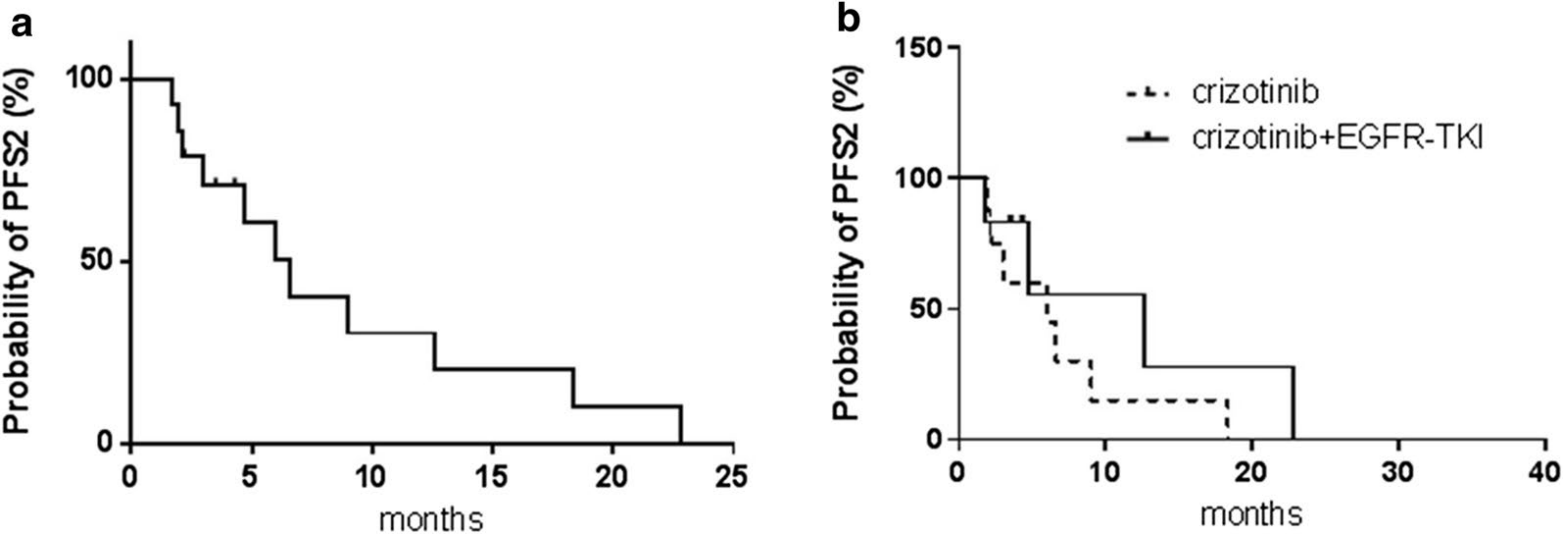
Progression-free survival of patients with acquired MET amplification treated with crizotinib after developing EGFR-TKI resistance. **a** 14 patients received crizotinib treatment, mPFS was 6.6 months, 95% CI 3.884–9.316; **b** for patients receiving crizotinib plus EGFR-TKI vs. crizotinib monotherapy, 12.6 vs. 6.0 months (P = 0.315)

A representative computed tomography (CT) scan (Fig. 1a, b) of one patient showed a remarkable response (adrenal metastases) after crizotinib treatment. The data regarding response duration with crizotinib treatment in 14 patients are presented in Fig. 2. The median PFS2 (mPFS2) for the 14 patients was 6.6 months (95% CI, 3.88–9.32 months; Fig. 3a), and for patients with or without EGFR-TKI treatment, the mPFS2 was 12.6 and 6.0 months, respectively (P = 0.315; Fig. 3b). In addition, eight of 14 patients and six of 14 patients had high- and intermediate-/low-level amplification; the mPFS2 for these two subgroups of patients was 6.6 and 6.0 months, respectively (P = 0.851).

No patient was lost during follow-up. Survival data were analyzed for 18 patients. The overall median OS (mOS) was 27.7 months (95% CI, 17.47–37.93 months). A significant difference existed between those patients treated with chemotherapy or crizotinib (12.0 months vs. 24.0 months, P = 0.046). In addition, the mOS for eight of 14 patients receiving crizotinib monotherapy and six of 14 patients receiving crizotinib plus EGFR-TKI was 17.2 and 24.0 months, respectively (P = 0.862).

### Safety and adverse events

Fourteen patients were included in the safety analysis of crizotinib treatment. The most common adverse events with crizotinib were vision disorders (most frequently, visual impairment, photopsia, or blurred vision), diarrhea, nausea, vomiting, and elevated liver aminotransferase levels. There was no edema, bradyarrhythmias, or QT prolongation. In the crizotinib monotherapy group, vision disorders occurred in two patients (25% [2/8]), elevated liver aminotransferase levels (12.5% [1/8]), and diarrhea (12.5% [1/8]); these events were grade 1 or 2. One patient experienced grade III nausea that resolved with crizotinib dose reduction to 200 mg twice daily (12.5% [1/8]). In the crizotinib with EGFR-TKI group, vision disorders (33.3% [2/6]), peripheral edema (16.7% [1/6]), and nausea and vomiting (16.7% [1/6]) were all grade 1 or 2. Two patients had grade 3 elevated liver aminotransferase levels (33.3% [2/6]) and received reduced doses of crizotinib (200 mg twice daily; Table 2).

**Table 2:** The summarize of treatment doses and toxicities for the 14 patients received crizotinib treatment after acquired resistance to first generation EGFR-TKI

Patient no.	Age/gender	*EGFR* mutataion	Treatment after acquired resistance	Grade 3–4 toxicities	Reduce dose
1	55/male	*21L858R*	Icotinib 125 mg/tid + crizotinib 250 mg/bid	No	No
2	65/female	*21L858R*	Gefitinib 250 mg/qd + crizotinib 250 mg/bid	Aminotransferase rise	Gefitinib 250 mg/qd + crizotinib 200 mg/bid
3	53/male	*21L858R*	Icotinib 125 mg/tid + crizotinib 250 mg/bid	No	No
4	49/male	*21L858R*	Gefitinib 250 mg/qd + crizotinib 250 mg/bid	No	No
5	62/female	*21L858R*	Elotinib 150 mg/qd + crizotinib 250 mg/bid	Aminotransferase rise	Elotinib 150 mg/qd + crizotinib 200 mg/bid
6	60/female	*19 exon deletion*	Icotinib 125 mg/tid + crizotinib 250 mg/bid	No	No
7	37/male	*19 exon deletion*	Crizotinib 250 mg/bid	No	No
8	64/male	*21L858R*	Crizotinib 250 mg/bid	No	No
9	71/female	*21L858R*	Crizotinib 250 mg/bid	Nausea	Crizotinib 200 mg/bid
10	64/female	*21L858R*	Crizotinib 250 mg/bid	No	No
11	58/male	*21L858R*	Crizotinib 250 mg/bid	No	No
12	41/female	*21L858R*	Crizotinib 250 mg/bid	No	No
13	58/female	*21L858R*	Crizotinib 250 mg/bid	No	No
14	53/female	*21L858R*	Crizotinib 250 mg/bid	No	No

## Discussion

Both crizotinib monotherapy and crizotinib plus EGFR-TKI treatment provided promising outcomes. This is the report with a relatively large sample size that evaluates the efficacy of crizotinib for the acquired MET amplification after EGFR-TKI therapy in Asian NSCLC patients.

MET amplification is one of the mechanisms that contributes to acquired resistance to EGFR-TKIs. According to previous reports, 5%–20% of patients with metastatic EGFR-mutated NSCLC develop acquired resistance to EGFR-TKIs through MET amplification [21–23]. Previous studies have reported patients with EGFR-mutant NSCLC and acquired MET amplification treated with MET inhibitors [24–29]. Crizotinib is an effective MET inhibitor for patients with MET amplification [17]; however, the outcomes of patients treated with crizotinib after developing resistance to EGFR-TKIs has not been determined.

In the current study we reported that the incidence of an acquired resistance mechanism due to MET amplification was higher in patients with an exon 21 L858R mutation (88.9%) than an EGFR exon 19 deletion (11.1%). Emergence of the T790M mutation is regarded as the most common mechanism of acquired resistance to EGFR-TKIs. The incidence of acquired T790M mutations differs between patients with exon 19 deletions and patients with exon 21 L858R mutations. Jenkins et al. [30] conducted T790M detection testing in the AURA (327 patients) and AURA2 trials (383 patients), which found that patients with exon 19 deletions are at a higher risk of developing T790M mutations than patients with L858R mutations (73% vs. 58%; P = 0.0002). Piotrowska et al. [31] conducted a similar study and reported that the corresponding rates of T790M mutations were 69% (94/137) and 30% (41/137), respectively. An observation trial involving a greater number of patients is expected to verify this trend.

The MET gene is a clinically relevant mutation that predicts the response to treatment of MET inhibitors. It is well-known that targeted therapy based on genetic testing improves the survival of cancer patients. In our study, four patients who received chemotherapy rather than crizotinib therapy had a significantly shorter mOS compared with patients who received crizotinib treatment (39.5 months vs, 17.0 months, P < 0.001). Hence, patients with acquired c-MET amplification may benefit from crizotinib treatment. A large sample prospective clinical study is needed for further evaluation.

In addition, the efficacy and survival of such patients treated with crizotinib monotherapy or crizotinib plus an EGFR-TKI are unclear. Met gene-mediated acquired resistance to EGFR-TKIs involves the activation of signaling pathways downstream from PI3K/mTOR [14, 32]. MET amplification is sensitive to treatment with MET inhibitors, including crizotinib or other MET-TKIs [33, 34], which supports the approach to combining EGFR-TKI with a MET inhibitor to overcome acquired resistance. Yoshimura et al. [25] reported the first case of successful crizotinib monotherapy in EGFR-mutant NSCLC that acquired MET amplification during erlotinib therapy and PR, but PFS was not observed. Previous studies have investigated the efficacy of INC280 (a c-MET inhibitor) in combination with gefitinib in treating NSCLC c-MET-positive patients with EGFR-TKI resistance, which resulted in an ORR of 29% and a DCR of 73%; the mPFS was 5.6 months [33]. It has been demonstrated that MET inhibitors in combination with EGFR-TKIs can overcome EGFR-TKI resistance mediated by aberrant c-Met activation in NSCLC preclinical models [35, 36]; however, the efficacy and survival data of such patients treated with crizotinib have rarely been published. One case report showed that the PFS for a patient treated with crizotinib plus gefitinib was 8.8 months [25]. Veggel et al. [37] reported that crizotinib treatment resulted in short-lasting responses in patients with EGFR mutation-positive NSCLC who acquired c-MET amplification after EGFR-TKI therapy (all 8 patients). Veggel et al. [37] showed a PR of 50% and a mPFS of 1.4 months. The patients were treated with crizotinib monotherapy (n = 2) or in combination with a first (n = 3) or third (n = 3) generation EGFR-TKI. In our study, the mPFS of patients treated with crizotinib plus an EGFT-TKI was longer than crizotinib monotherapy (12.6 months vs. 6.0 months, P = 0.315). We believe the efficacy of crizotinib may be involved with the co-occurrence of other gene mutations, therapeutic models, and the cMET-to-CEP7 ratio.

In our study, two patients (33.3% [2/6]) had grade 3 adverse events and received dose adjustments. There is only one phase 1 study which reported 100 mg and 150 mg twice a day as the MTD for erlotinib and crizotinib, respectively [38]. To date, no standard combination treatment regimens have been established. Therefore, the patients received a standard dose of crizotinib whether in the crizotinib alone or combination treatment group. We believed that patients who had already been treated with a EGFR-TKI might be better able to tolerate adverse reactions than patients who were initially treated with an EGFR-TKI and crizotinib combination. Veggel et al. [37] reported that a crizotinib dose modification was necessary for three patients, two of whom were treated with crizotinib monotherapy and one of whom was treated with crizotinib and osimertinib. Among the three patients who were treated with crizotinib plus erlotinib, all patients tolerated the standard dose with no dose adjustment. Therefore, patients with an acquired MET, combination therapy did not increase the frequency of adverse reactions and the patients tolerated standard doses. In addition, combination of the two drugs may increase hepatic toxicity. Icotinib was associated with a lower number of treatment-related adverse events when compared to gefitinib with respect to overall incidence and diarrhea, suggesting that icotinib might have a better safety profile. Icotinib has a better safety profile than gefitinib for other major adverse events [39]. Furthermore, high-dose icotinib (250 mg tid) has tolerable toxicity in NSCLC patients harboring 21 L858R mutations [40]. Hence, icotinib is relatively well-tolerated, and adverse reactions are controllable in patients receiving combination therapy. Of course, in clinical treatment, we must closely monitor adverse reactions and make drug dose adjustments in a timely manner.

The limitations of our study should be mentioned. Despite the fact that the present study recruited the largest patient sample compared with similar previous studies, only six of 18 patients were treated with crizotinib plus an EGFR-TKI. The power of the statistical analysis may have been compromised due to the limited group members. Furthermore, other potentially existing mutations were not considered.

## Conclusion

EGFR-mutated NSCLC patients developed resistance to EGFR-TKIs due to MET amplification with EGFR-TKI plus c-Met inhibitors by providing simultaneous inhibition of both pathways. Patients with acquired MET amplification benefited from crizotinib monotherapy and crizotinib plus EGFR-TKI treatment. Tolerance to combination therapy was acceptable, but requires close monitoring. Randomized trials or well-designed clinical trials are needed in the future to establish the role of crizotinib in patients with acquired resistance.
